# A Simple Turn-off Schiff Base Fluorescent Sensor for Copper (II) Ion and Its Application in Water Analysis

**DOI:** 10.3390/molecules26051233

**Published:** 2021-02-25

**Authors:** Xing Zhang, Ling-Yi Shen, Qi-Long Zhang, Xian-Jiong Yang, Ya-Li Huang, Carl Redshaw, Hong Xu

**Affiliations:** 1 The key Laboratory of Environmental Pollution Monitoring and Disease Control, School of Public Health, Ministry of Education, Guizhou Medical University, Guiyang 550004, China; zhangxing11207115@126.com; 2 School of Basic Medical Science, Guizhou Medical University, Guiyang 550004, China; shenly@stumail.nwu.edu.cn (L.-Y.S.); yangxianjiong@126.com (X.-J.Y.); ylh6401@gmc.edu.cn (Y.-L.H.); 3 Department of Chemistry, University of Hull, Cottingham Road, Hull, Yorkshire HU6 7RX, UK; c.redshaw@hull.ac.uk

**Keywords:** synthesis, fluorescent probe, copper(II) ion, AIEE, water detection

## Abstract

An aniline-functionalized naphthalene dialdehyde Schiff base fluorescent probe **L** with aggregation-induced enhanced emission (AIEE) characteristics was synthesized via a simple one-step condensation reaction and exhibited excellent sensitivity and selectivity towards copper(II) ions in aqueous media with a fluorescence “ turn-off ” phenomenon. The detection limit of the probe is 1.64 × 10^−8^ mol·L^−1^. Furthermore, according to the results of the UV-vis/fluorescence titrations, Job’s plot method and ^1^H-NMR titrations, a 1:2 stoichiometry was identified. The binding constant between **L** and Cu^2+^ was calculated to be *K_a_* = 1.222 × 10^3^. In addition, the AIEE fluorescent probe **L** could be applied to detection in real water samples with satisfactory recoveries in the range 99.10–102.90% in lake water and 98.49–102.37% in tap water.

## 1. Introduction

In recent years, fluorescent probes have been widely used in the field of rapid detection (food, metal ion, anion, biomarker, etc.) due to advantages such as ease of operation, low cost, high selectivity, and sensitivity [1,2,3,4]. Copper is an earth abundant element which plays an important role in many domains like industrial material, [5] bactericide, and herbicide [6,7], etc. On the other hand, copper ions are important environmental pollutants. It not only produces toxic effects on the growth of animals and plants, but also lead to risks to human health via the food chain [8,9,10]. Lack or excess of copper will cause health damage.

In order to protect human health, the WHO stipulates that the limit of copper ions in drinking water should not exceed 31.4 μM [7]. Thus, the development of technologies for copper ion detection has become an important focus in environmental and food nutritional fields. Traditional methods such as atomic absorption spectroscopy (AAS), gas chromatography-mass spectrometry (GC-MS), inductively coupled plasma mass spectrometry (ICP-MS) and high performance liquid chromatography (HPLC) have been widely utilized for detection copper ion [3,11], but these systems also have their limitations, e.g., large and expensive instruments, complex modes of operation and high detection costs. In recent years, methods based on fluorescent probes have emerged as the more cutting-edge technology in the field of metal ion detection due to convenient procedures, super sensitivity, and fast response times [12,13]. Many copper(II) probes have been reported due to their low detection limit and remarkable optical changes [14,15].

However, most traditional fluorescent materials exhibit an aggregation caused quenching (ACQ) effect, which means they are highly emissive in solution, but a quenching affect exists in the aggregation state. In the case of dyes, the ACQ effect can greatly limit their practical applications in material science [16]. In contrast, an abnormal photophysical phenomenon was observed by Tang’s group in 2001 [2,17], namely, aggregation induced emission (AIE) and aggregation-induced emission enhancement (AIEE), whereby the fluorescent molecules show non-/weak emission in solution but enhanced fluorescence in the aggregation state. Molecules emit energy through intramolecular rotation and vibrational motions in the solution, while in the aggregation state the non-radiative transition is blocked, and, in turn, energy is lost through the radiative transition channel [18].

Naphthalene and its derivatives have been extensively applied in the assembly of light-emitting devices and chemical sensors due to their excellent optical properties and unique chemical stability [19,20,21]. In this research, we have developed a small molecular probe **L** possessing AIEE characteristics via a simple one-step reaction, which has good recognition performance and anti-interference ability for copper ion detection under aqueous conditions.

## 2. Results and Discussion

### 2.1. Synthesis

**Scheme 1:**
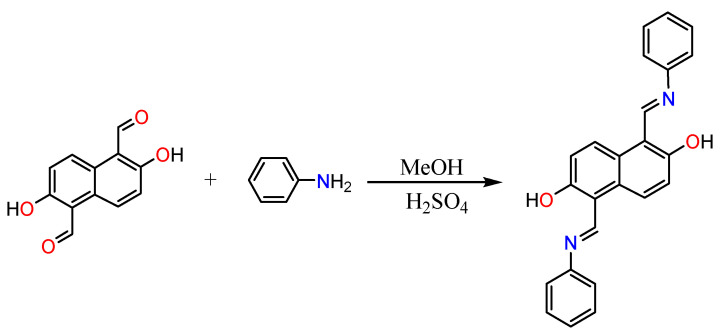
Synthetic route to Schiff-base **L**.

A new Schiff base probe **L** was obtained from 2,6-dihydroxynaphthalene-1,5-dialdehyde and aniline by a simple one-step reaction. The molecular structure was characterized by ^1^H-NMR spectroscopy, HRMS, and single crystal X-ray diffraction. The Schiff base probe **L** exhibited excellent solubility in common solvents (such as dichloromethane, tetrahydrofuran, DMSO, etc.) and possessed good acid and alkali-resistance over the pH range 3–11 within 1440 min (Table S2). This work provides a new strategy for extending the practical applications of small molecular probes in the heavy metal detection field. (Scheme 1).

### 2.2. AIEE Properties

**Figure 1:**
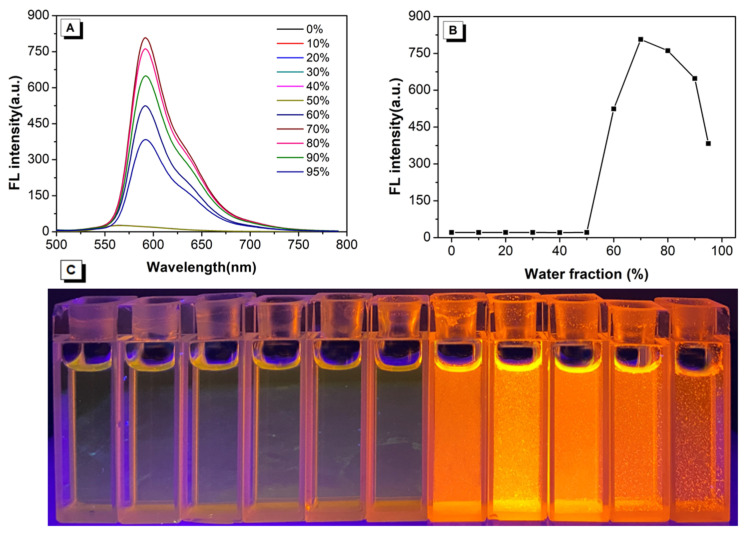
(**A**) Fluorescence spectra of **L** (40 μM) in tetrahydrofuran (THF)/water mixtures with different water fractions (λ_ex_/λ_em_ = 428 nm/565 nm, slit: 5/5 nm, voltage: 660 v). (**B**) Plots of fluorescence intensity at 565 nm. (**C**) photographs in THF/water mixtures with different water fractions taken under 365 nm UV irradiation.

The probe **L** emits glaring red light in the solid state but the emission decreases dramatically in the dissolved state, so we propose that this probe may exhibit AIEE properties. To investigate our hypothesis, a good solvent tetrahydrofuran (THF) and a bad solvent water were selected as the testing systems. As shown in Figure 1, the compound **L** emitted orange mission with λmax em = 565 nm in pure THF solution. As the water fraction (*f*w) gradually increased from 0% to 50%, the fluorescence intensity barely changed. Subsequently, when the water fraction (*f*w) reached 60%, some particles could be observed in the mixture, and the mixture exhibited bright orange light under 365 nm UV irradiation. The fluorescence spectrum also corresponded with this phenomenon: the fluorescence intensity at 60% was much higher than that of *f*w = 0–50%. When the water fraction reached 70%, the fluorescence intensity of the solution attained the maximum value (807 a.u.) with an approximate 40.31-fold increase versus that in the pure solution. In addition, we also tested the quantum yield of **L** in the solid state and in pure THF solvent. The quantum yield in the solid state (Φ *f* = 18.4%) is higher than that in THF solution (Φ *f* = 0.5%). Thus, the Schiff-base **L** is a chromophore with aggregation-induced enhanced emission characteristics. As the probe polymerizes at *f*w = 60%, referring to the experimental conditions of similar probes [20,22], we choose the mixture of THF/water (V_THF_/V_H2O_ = 4:1) as the recognition environment.

### 2.3. Stability of Detection

The luminescent and recognition behavior of Schiff base compounds are often affected by many factors (such as pH, time, etc.). If the recognition environment tends to acidic/alkaline, this can induce C=N fracture, which may affect the optical properties and recognition ability of these compounds [23,24]. In order to test the luminescent stability of probe **L**, the pH 3–11 range was selected as the detection environment. In addition, the pre-experimental results showed that the probe **L** had the ability to recognize Cu^2+^, so the mixed solution of probe **L**-Cu^2+^ was also selected for determination at the same time.

**Figure 2:**
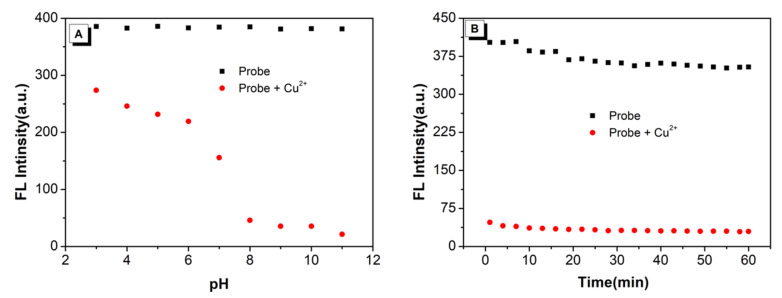
The fluorescence intensity stability of the **L** and **L**-Cu^2+^ systems versus different (**A**) pH values and (**B**) reaction time.

As shown in Figure 2A, the emission intensity of probe **L** at 565 nm barely changed, which indicated that this probe possessed good acid and alkali-resistance over the pH range 3–11. When adding Cu^2+^ to the mixture, a significant fluorescent quenching phenomenon was observed over the pH range from 8 to 11, whilst little fluorescence change was found over the pH range 3 to 7. The acidic conditions can induce dissociation of the **L**-Cu^2+^ complex because of the protonation of probe, and excessive H^+^ in the solution may push the coordination equilibrium to the left [25]. As for higher pH conditions, the alkaline recognition environment can provide sufficient hydroxyl ions to consume H^+^, and the ionized phenolic groups interacted more easily with copper ions to form **L**-Cu^2+^ complex [26,27]. These may be the reasons why probe **L** exhibited better recognition ability under alkaline conditions. We also tested the time-dependent optical stability of probe **L** and the **L**-Cu^2+^ mixture, and the results showed that **L** and the **L**-Cu^2+^ complex were stable over a certain period of time.

### 2.4. Cation Sensing Study

Schiff base compounds are widely used in the recognition of rare-earth metals, transition metals and alkali metals as a result of their high binding affinity [28,29,30]. To test the detecting ability toward metal ions, probe **L** (40 μM) was exposed to many metal ions (such as Cd^2+^, Cu^2+^, K^+^, Pb^2+^, Li^+^, Fe^3+^, Mg^2+^, Co^2+^, Cr^3+^, Al^3+^, Ba^2+^, Ni^2+^, Zn^2+^, Ag^+^, Na^+^, Hg^+^, [M]^n+^ = 200 μM) and anions (such as AcO^−^, ClO_4_^−^, Br^−^, NO_2_^−^, CO_3_^2−^, I^−^, F^−^, SO_4_^2−^, Cl^−^, PO_4_^3−^, C_2_O_4_^2−^, SO_3_^2−^, HSO_3_^−^, HCO_3_^−^, [A]^n−^ = 200 μM) in mixtures of THF and water (V_THF_/V_H2O_ = 4/1, pH = 8.00).

**Figure 3:**
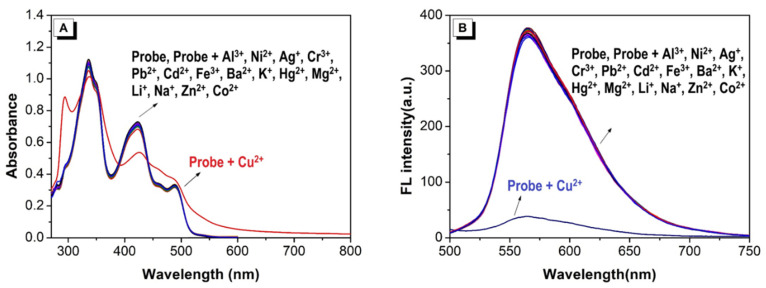
(**A**) The UV–vis and (**B**) Fluorescence spectra of the fluorescence probe **L** interacting with different metal ions (λ_ex_/λ_em_ = 428/565 nm, slit: 5/5 nm, voltage: 900 v).

**Figure 4:**
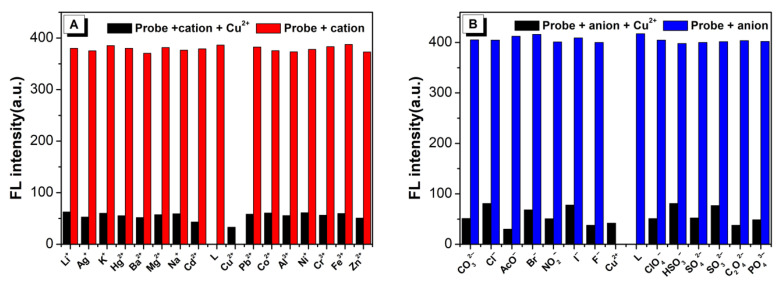
Bar diagram of the competitive experiments of various metal cations (**A**) and anions (**B**) on the fluorescence intensity of the probe/Cu^2+^ complex in buffer solution.

As shown in Figure 3, on adding the metal ions to the solvent containing **L**, only Cu^2+^ cause the solution colour to change via naked-eye observation (Figure S3). The absorption spectra and fluorescence spectra of **L–**cation mixture indicated that probe **L** exhibits good selectivity toward Cu^2+^, while other cations or anions (Figure S4) had little impact on the optical behavior of probe **L**. On other hand, under a 365 nm UV lamp, only the **L**-Cu^2+^ mixture led to the emission light quenching dramatically (Figure S3). Furthermore, competitive experiments were also performed to investigate the selectivity of the probe toward Cu^2+^. When Cu^2+^ was present in the solution, the emission of the mixture at λ_em_ = 565 nm was quenched, while without Cu^2+^, the emission barely changed (Figure 4), which suggested that the coexisting ions/anions had a limited impact on the detection of Cu^2+^. Thus, the interference experiments indicated that the probe displays a high specificity and selectivity for detecting Cu^2+^ ions.

### 2.5. Job’s Plot and Binding Constant 

**Figure 5:**
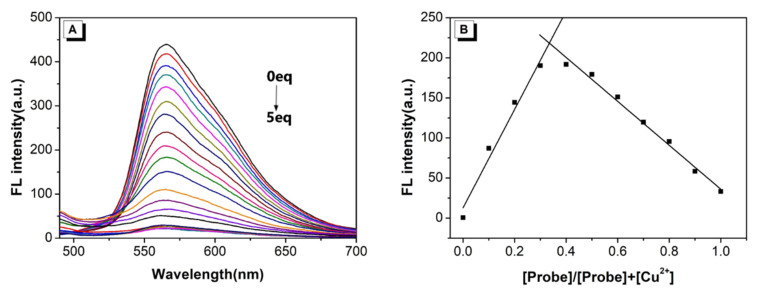
(**A**) Fluorescence spectra on addition of Cu^2+^ to the probe; (**B**) Job’s plot for the determination of the stoichiometry of **L** and Cu^2+^ in the mixture of THF/H_2_O (V_THF_:V_H2O_ = 4/1), the total concentration of **L** and Cu^2+^ was 40 μM.

Based on the above experimental conditions, the fluorescence titration experiments were performed with progressive addition of Cu^2+^ and are presented in Figure 5. As the figure demonstrates, the fluorescence intensity of probe **L** at λ_max em_ = 565 nm gradually decreased as the Cu^2+^ ions were added. In addition, when the concentration of probe **L** change from 0 to 72μM (Figure S5), there exist a good linear relationship between the probe and the copper(II) ions (y = 432.59482 − 218.198x, R^2^ = 0.99276). Herein, the detection limit was calculated by utilizing the data of the fluorescence titration experiments following the IUPAC method (IUPAC Compendium of Analytical Nomenclature): 10 groups of blank samples were tested in the absence of copper under the same conditions, and then the standard deviation (SD) was calculated from the emission peak at 565 nm. After that, following the formula: the detection limit = 3SD/S, where S is the slope of the linear relationship during the fluorescence titration, the detection limit of probe **L** for Cu^2+^ is calculated to be 16.4 nM. Comparing with other Cu^2+^ probes (Table S3), the probe **L** has the advantages of lower detection limit and simpler synthetic route.

**Figure 6:**
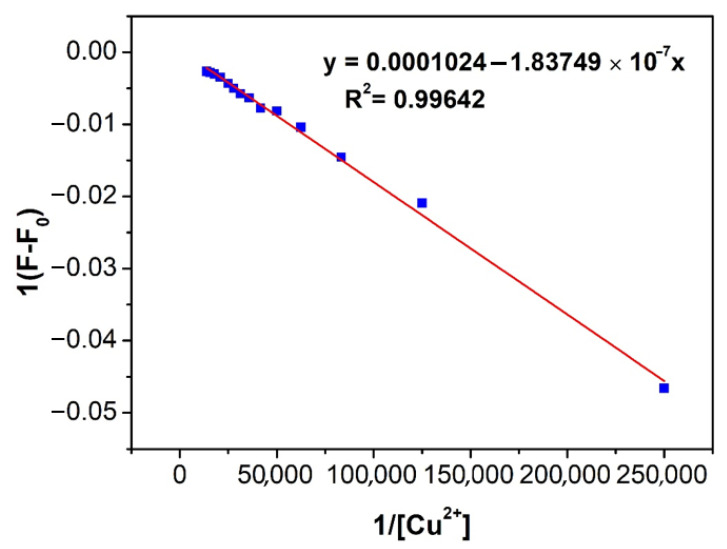
The Benesi–Hildebrand plot of 1/(F − F_0_) versus 1/[Cu^2+^].

Further, the binding mode of probe **L** and Cu^2+^ was investigated by a Job’s plot by controlling the total concentration of the probe and Cu^2+^ at 40 μM in a mixture of THF/H_2_O (V_THF_:V_H2O_ = 4/1). The results indicated that the binding stoichiometry between the probe and Cu^2+^ was 1:2. On the other hand, a Benesi–Hildebrand (Figure 6) curve based on the above experiments could been obtained and the binding constant calculated. According to the formula [31]:

F − F_0_ = △F = [Cu^2+^](F_max_ − F_0_)/(1/K_a_ + [Cu^2+^]) (1)

Here, F and F_0_ are the corresponding fluorescence intensity at 598 nm in the presence and absence of Cu^2+^, and F_max_ is the fluorescence intensity when the probe has completely complexed with cations. Based on the data of fluorescence titration and the slop of the correlation curve, the binding constant (K_a_) was calculated to be K_a_ = 1.222 × 10^3^.

### 2.6. A Possible Mechanism for Detection Cu^2+^

**Figure 7:**
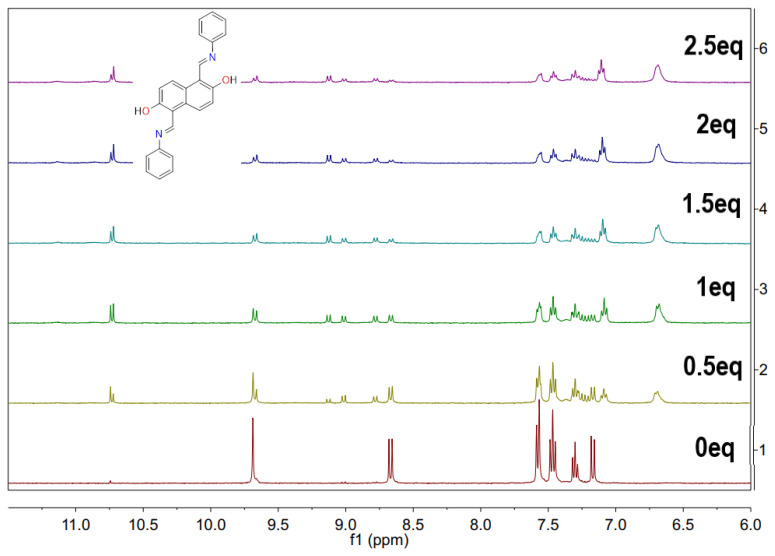
^1^H-NMR spectroscopic titration spectra of **L**-Cu^2+^ on increasing concentrations of Cu^2+^ in d-DMSO/D_2_O solution.

To better understand the recognition mechanism of **L** for detecting copper ions, ^1^H-NMR spectroscopic titration experiments in d-DMSO: D_2_O solution were performed. As shown in Figure 7, when there did not exist any copper ions in the solution, one single peak (δ = 9.69 ppm) and one doublet (δ = 8.66–8.68 ppm) were observed corresponding to the -OH and -CH=N, respectively. The proton peaks at δ = 7.16–7.59 ppm were from the naphthalene ring and aniline ring of the probe **L**. When copper ions were added into the mixture, the proton signals at 9.69 ppm and 8.66 ppm were weakened and split, and new proton peaks at 10.72–10.74 ppm, 9.00–9.03 ppm, and 9.12–9.14 ppm could be observed at the same time. This indicated that in the presence of Cu^2+^, the O atom from the hydroxyl and the N atom from the -CH=N bond coordinated with Cu^2+^ firstly. On the other hand, as the initial content of probe **L** exceeded that of the Cu^2+^ in the mixture, there existed two coordination sites in one **L** molecule, and the probe molecule adopted a pattern with partially complexed with Cu^2+^. Thus, the proton environment of the -OH and -CH=N exhibited differences at different ratios of **L** and Cu^2+^, respectively. Similarly, the proton signals from the naphthalene and benzene rings also undergo a high field shift from 7.16–7.59 ppm to 7.09–7.57 ppm, indicating that the probe **L** does interact with Cu^2+^. On further increasing the amount of Cu^2+^ (1eq–2.5eq), the proton signals of -OH and -CH=N became much weaker and tend to be stable after the content of Cu^2+^ reached 2.0 eq, suggested that the probe **L** and Cu^2+^ may adopt a 1:2 coordination mode, consistent with the results obtained via the Job’s plot method.

**Figure 8:**
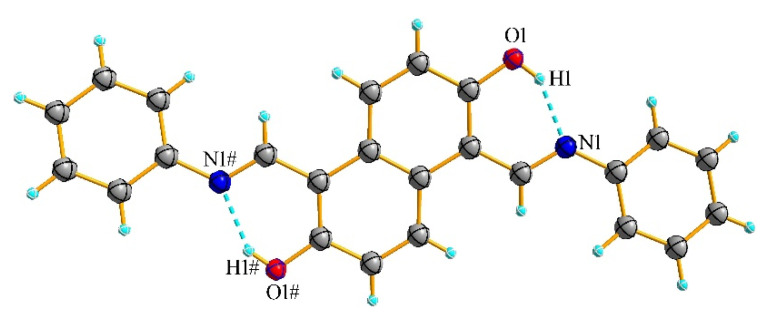
The X-ray single crystal diffraction image of probe L (Dashed lines represent hydrogen bonds, Symmetry code:# = −*x*, 1 − *y*, 1 − *z*).

**Figure 9:**
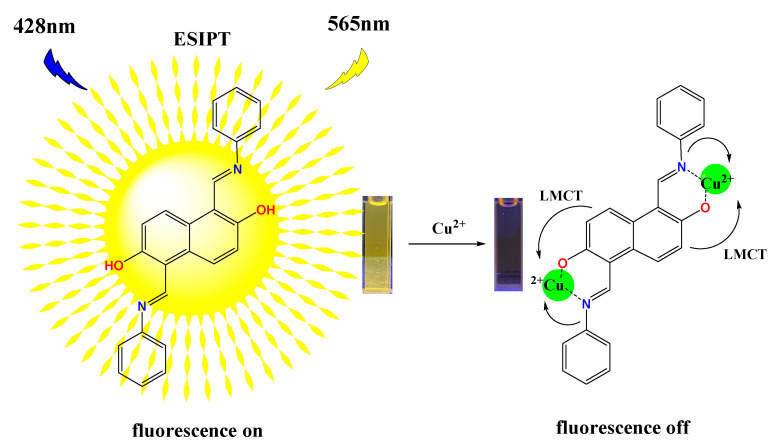
The possible recognition mechanism process for detection of copper ions.

The data from the single crystal X-ray diffraction analysis verify that two benzene rings at the ends of the probe are parallel to each other, but the naphthalene ring is not parallel to the benzene rings. As shown in Figure 8, the dihedral angle between the N1/N# (connecting with the plane of benzene) and the double bond C (connecting with the plane of naphthalene) is 167.14°, so probe **L** is a nonplanar molecule. Furthermore, the hydrogen atoms on two hydroxyl groups form a stable six membered ring structure with the N atoms from imine groups via the O-H···N intramolecular hydrogen bonds. As the 2,6-dihydroxynaphthalene-1,5-dialdehyde reacts with aniline to form the -CH=N bond, this functional group bridges the naphthalene and benzene ring leading to the probes conjugated structure. When probe molecules are excited by light, an electron flow from the naphthalene ring to the imine group can easily occur; on the other hand, in the THF/H_2_O solution, there exist a fast structure convert from enol form to keto form in the molecule [32], the intramolecular hydrogen bonds limit the nonradiative transition; thus, the probe **L** in the dissolved state emits orange light [33,34,35]. According to the ^1^H-NMR spectroscopic titration experiments, the probe coordinates with Cu^2+^ via the N atom of imine and the O atom of -OH to form a stable complex. At this time, the lone pair electrons on the N and O atoms will transfer to the empty orbitals of the copper ion, which blocks the initial excited electron transfer process (Figure 9) [36], resulting in fluorescence quenching of the probe solution.

### 2.7. Applications

In order to further evaluate the potential application of probe **L** for the detection of Cu^2+^ in real specimens, water samples from an artificial lake (at Guizhou Medical University) and running water (at our laboratory) have been collected for testing. The specific experimental process is as follows: 7.6 mL THF solution, 400 μL probe stock solution (40 μM), 1 mL buffer solution, and 1 mL water sample (had been filtered) were added into one volumetric flask and the mixture shaken well. At the same time, another water sample was processed with the same steps and an appropriate amount of standard substance (Cu(NO_3_)_2_) was added in it. After standing for 30 min, the fluorescence intensity of the sample at 565 nm had been recorded for further calculations. As shown in Table 1, the recoveries of the probe were calculated in the range of 99.10–102.90% in lake water and 98.49–102.37% in tap water. These results suggest that **L** is a sensitive and selective probe for Cu^2+^ monitoring in environmental water samples.

## 3. Experimental Section

### 3.1. Materials and Characterization

Unless otherwise stated, all of the starting materials were commercially available and used without further purification. The solution of the metal ions and anions were prepared from their nitrate and sodium salts, respectively. ^1^H-NMR spectra (400 MHz) were recorded on a Inova-400 Bruker AV 400 spectrometer (Bruker, Karlsruhe, Germany) using d-DMSO solvent and tetramethylsilane as the internal reference. J-values are given in Hz. High resolution mass spectra (HRMS) were recorded on a GCT premier CAB048 mass spectrometer (Waters Corp., Milford, MA, USA) operating in a MALDI-TOF mode. UV-vis absorption spectra were obtained on a UV-2600 Milton Ray Spectrofluorometer (Shimadzu, Kyoto, Japan). PL spectra were recorded on a Cary Eclipse Hitachi 4500 spectrofluorometer (Hitachi, Tokyo, Japan). The crystal structure was determined by Bruker Smart Apex single crystal diffractometer (Bruker, Karlsruhe, Germany).

### 3.2. Synthesis of the Compound L

Firstly, 43.2 mg (0.2 mmol) 2,6-dihydroxynaphthalene-1,5-dialdehyde was dissolved in 30 mL methanol, and then 50 μL (95%, 5.44 mmol) aniline was added into the mixture and stirred for 10 min in room temperature. After that, adding 10 μL H_2_SO_4_ into the mixture and stirring for another 5 h. The precipitate was filtered and washed with hot methanol for three times, and dried in vacuum to obtain the target red compound (63 mg, 0.17 mmol, yield 86.12%). An appropriate amount of precipitate was dissolved in a mixture of tetrahydrofuran and methanol (2:1), and then evaporated. After 4 days, red acicular crystals suitable for single crystal diffraction analysis were obtained. ^1^H-NMR (400 MHz, DMSO, TMS) δ 9.69 (s, 2H), 8.67 (d, *J* = 9.4 Hz, 2H), 7.58 (d, *J* = 7.8 Hz, 4H), 7.47 (t, *J* = 7.7 Hz, 4H), 7.30 (t, *J* = 7.3 Hz, 2H), 7.17 (d, *J* = 9.4 Hz, 2H),. HRMS calculated: 398.11, found 398.15.

### 3.3. X-ray Crystallography

Crystallographic data for ligand **L** was collected on a Bruker APEX 2 CCD diffractometer with graphite-monochromated Mo Kα radiation (λ = 0.71073 Å) in the ω scan mode [37]. The structure was solved by a charge flipping algorithm and refined by full-matrix least-squares methods on F2 [38]. All relative standard deviation were estimated using the full covariance matrix. Further details are presented in Table S1. CCDC: 2059923, **L**.

### 3.4. General Methods for Optical Tests

Firstly, 3.7 mg (10μM) of probe **L** was dissolved in 10.00 mL THF solution to prepare 1mM stock solution. Then the nitrate of metal ions and the sodium salt of anions (Ag^+^, Al^3+^, Ba^2+^, Cd^2+^, CO^2+^, Cr^3+^, Na^+^, Cu^2+^, K^+^, Ni^2+^, Pb^2+^, Zn^2+^, Hg^2+^, Fe^3+^, Mg^2+^, Li^+^, AcO^−^, ClO_4_^−^, Br^−^, NO^2−^, CO_3_^2−^, I^−^, F^−^, SO_4_^2−^, Cl^−^, H_2_PO_4_^−^, PO_4_^3−^, HPO_4_^2−^, C_2_O_4_^2−^, SO_3_^2−^, HSO_3_^−^, HCO_3_^−^) were accurately weighed and dissolved in 10.00 mL Tris-HCl buffer to form 10 mM ion stock solution. The preparation method of Tris-HCl buffer solution (2 mM) was as follows: 121.20 mg of trimethylol aminomethane was dissolved in 1.00 L ultrapure water, then its pH was adjusted to 8.00 with 0.10 M HCl solution and 0.10 M NaOH solution.

## 4. Conclusions

In summary, we have developed a new simple AIEE fluorescent probe based on two aniline units attached to a naphthalene dialdehyde core. The fluorescence intensity of the probe in good solvents and poor solvents can differ by 40.31 times. Furthermore, in the presence of copper(II) ions, the probe solution showed an obvious colour change from yellow to brown under daylight and from bright to dark under UV lamp irradiation with a detection limit as low as 1.64 × 10^−8^ mol·L^−1^. This indicates that the probe **L** has the potential to be used for the detection of Cu^2+^ by naked eye and via instrumentation. Based on the titration experiments, a good linear relationship was found which may apply the probe to the quantitative and qualitative detection of Cu^2+^ in real samples. We believe this work not only provides a new example of a small molecular probe for ion detection, but these results may inform research in broader fields such as biometrics and photodynamic therapy, and such research is going-on in our laboratory.

## Figures and Tables

**Table 1:** The detail data for Cu^2+^ detection in real water samples.

Sample	Measured	Added	Detected	Recovery	RSD
	(μmol·L^−1^)	(μmol·L^−1^)	(μmol·L^−1^)	(*n* = 3, %)	(*n* = 3, %)
**Running water**	8.99	5.00	14.03	99.71	0.93
		10.00	19.28	98.49	2.01
		20.00	28.32	102.37	1.15
**Artificial lake**	15.92	5.00	21.11	99.10	1.48
		10.00	25.19	102.90	1.72
		20.00	35.99	99.81	2.07

## Data Availability

No new data were created or analyzed in this study. Data sharing is not applicable to this article.

## Supplementary Materials

The following are available online, Figure S1: ^1^H-NMR of probe **L** title, Figure S2: HRMS spectrum of **L**, Figure S3: Photographs of probe **L**-cation complex under natural light and 365 nm UV lamp, Figure S4: UV-vis and Fluorescence spectra of the fluorescence probe **L** interacting with different anions; Photograph of probe **L**-anions complex in THF/water (V_THF_/V_water_ = 4/1, pH = 8.00) solution under 365 nm UV lamp, Figure S5: The absorbance and fluorescence spectra on addition of Cu^2+^ to the probe (40 μM, V_THF_:V_H2O_ = 4/1, λ_ex_ = 428 nm, slit: 5/5 nm, voltage: 900 v). The fluorescence intensity change plots at 565 nm on addition of Cu^2+^. Insert: when [Cu^2+^]/[probe] is in the range of 0–1.8 ratio, the fluorescence intensity of the probe has a good linear relationship with Cu^2+^ (y = 432.59482 − 218.198x, R^2^ = 0.99276), Table S1: Summary of crystal data of probe **L**, Table S2: The fluorescence intensity(a.u.) of probe L and L-Cu^2+^ complex versus different pH value within 1440 min (565 nm), Table S3: Comparison data with reported Cu^2+^ sensors.
