# Supplementary material for: A Simple Turn-off Schiff Base Fluorescent Sensor for Copper (II) Ion and Its Application in Water Analysis
Source: Molecules. 2021 Feb 25;26(5):1233. doi: 10.3390/molecules26051233 (PMC7956479; doi:10.3390/molecules26051233)
Supplement: Supplementary file 1 [file molecules-26-01233-s001.pdf]

Supporting Information

A Simple Turn-off Schiff base Fluorescent Sensor for Copper(II) Ion and its Application in Water Analysis

Xing Zhang [1], Ling-yi Shen[2], Qi-Long Zhang [1,2,*], Xian-Jiong Yang[2], Ya-Li Huang[2], Carl Redshaw [3], Hong Xu[1,2,*]

[1] School of Public Health, the key Laboratory of Environmental Pollution Monitoring and Disease Control, Ministry of Education, Guizhou Medical University, Guiyang 550004, China. Email: zhangxing11207115@126.com (X.Z)

[2] School of Basic Medical Science, Guizhou Medical University, Guiyang 550004, China. Email: shenly@stumail.nwu.edu.cn (L.Y.S); sciqlzhang@gmc.edu.cn (Q.L.Z) ; yangxianjiong@126.com (X.J.Y); ylh6401@gmc.edu.cn (Y.L.H); xuhong@gmc.edu.cn (H.X.).

[3] Department of Chemistry, University of Hull, Cottingham Road, Hull, Yorkshire HU6 7RX, UK. mail: c.redshaw@hull.ac.uk

* Correspondence: xuhong@gmc.edu.cn(H.X.); Fax: +86-851-8817-4017 (H.X.); gzuqlzhang@126.com or sciqlzhang@gmc.edu.cn(Q.L.Z.); Fax: +86-851-8817-4017 (H.X.)

Contents:

^{1}H NMR spectrum of probe L **S1**

HRMS spectrum of L **S2**

Photophysical properties of L **S3-S5**

X-ray crystallographic analysis of L **Table S1**

Comparison data with reported Cu^{2+} sensors **Table S2**

NMR spectrum

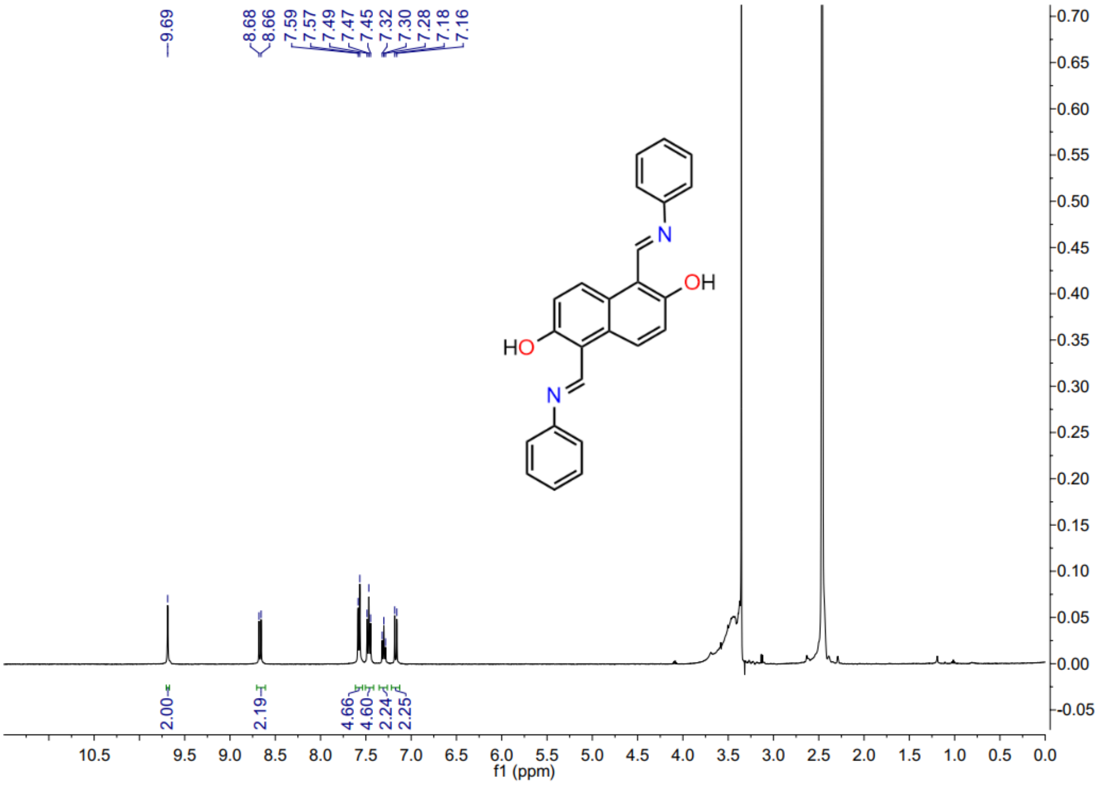

Fig. S1. ^{1}H NMR of probe **L**

High Resolution Mass Spectra

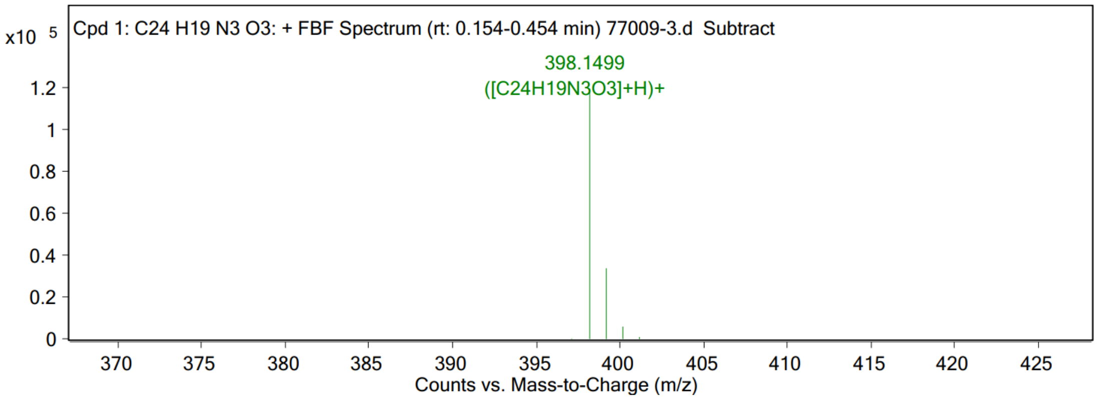

Fig. S2. HRMS spectrum of **L**

Photophysical Properties

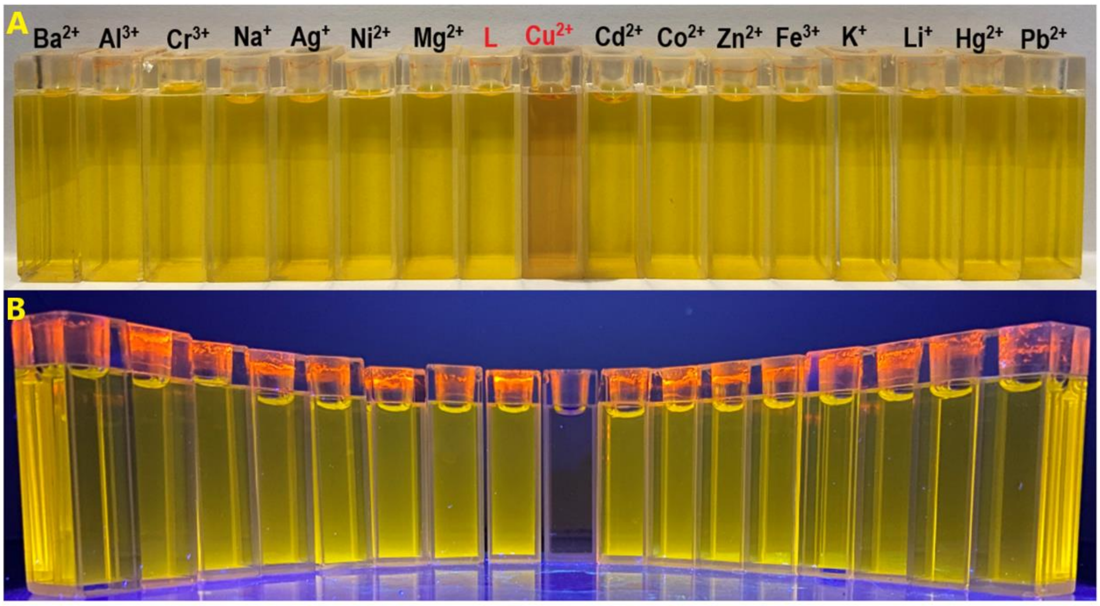

Fig. S3. Photographs of probe **L** - cation complex in THF/water (V_{THF}/V_{water}=4/1, pH=8.00) solution under (B) natural light and (C) 365 nm UV lamp.

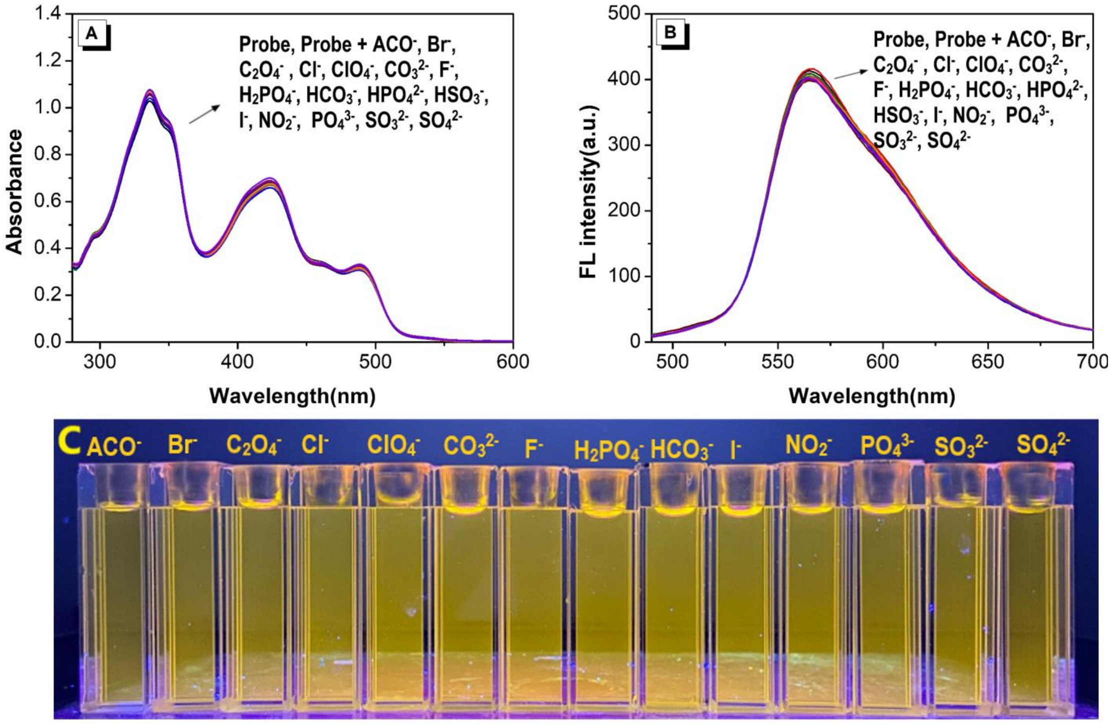

Fig. S4. (A) UV-vis and (B) Fluorescence spectra of the fluorescence probe **L** interacting with different anions ($\lambda_{ex}/\lambda_{em}$ = 428/565 nm, slit: 5/5nm, voltage: 900 v). (C) Photograph of probe **L** - anions complex in THF/water (V_{THF}/V_{water}=4/1, pH = 8.00) solution under 365 nm UV lamp.

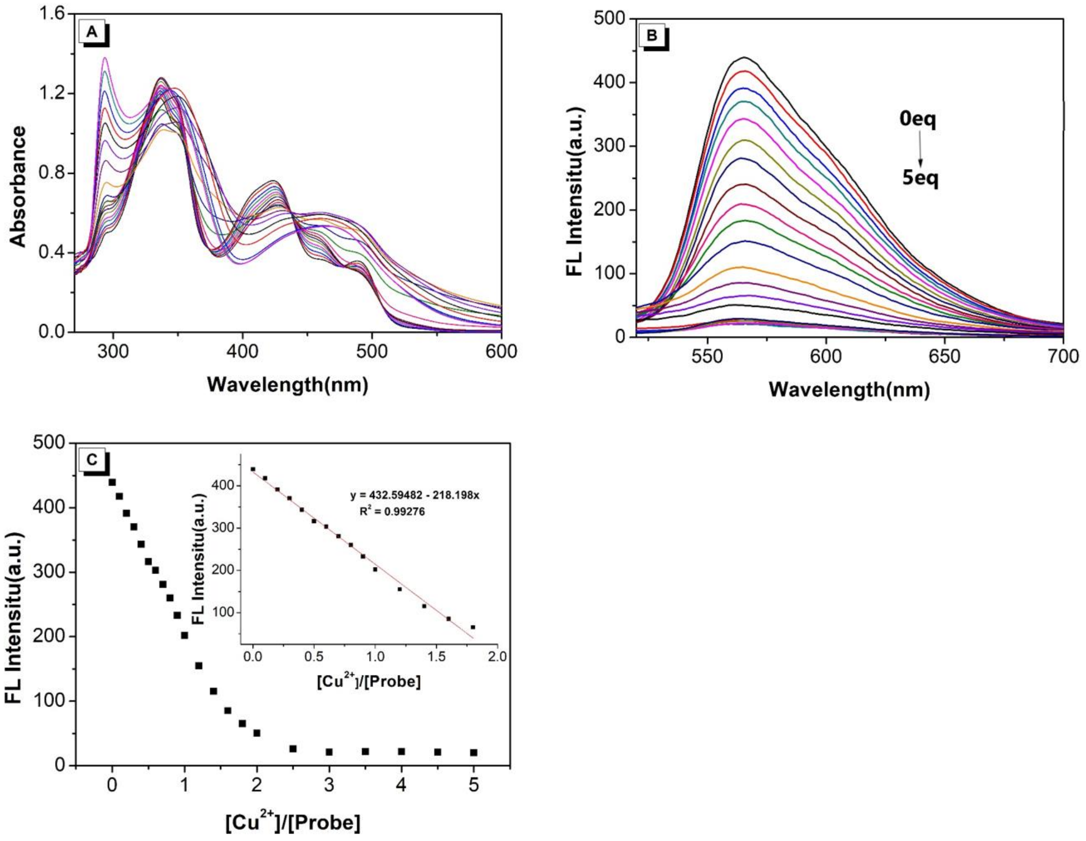

Fig. S5. (A) The absorbance and (B) fluorescence spectra on addition of Cu^{2+} to the probe (40μM, V_{THF}:V_{H2O} = 4/1, λ ex = 428 nm, slit: 5/5 nm, voltage: 900 v). (C) The fluorescence intensity change plots at 565 nm on addition of Cu^{2+}. Insert: when [Cu^{2+}] / [probe] is in the range of 0 - 1.8 ratio, the fluorescence intensity of the probe has a good linear relationship with Cu^{2+} (y=432.59482 – 218.198x, R^2=0.99276).

X-ray crystallography Analysis

Table S1. Summary of crystal data of probe **L**

Parameter	Probe
Empirical formula	$C_{24}H_{18}N_2O_2$
Formula weight[g mol^{-1}]	366.40
Crystal system	monoclinic
Space group	$P2_1/n$
a [Å]	4.9480(5)
b [Å]	17.867(2)
c [Å]	10.1149(12)
β [°]	93.732(4)
Volume [Å^3]	892.34(17)
Z	2
Density, calcd [gm^{-3}]	1.364
Temperature [K]	273(2)
F(000)	384.0
Unique reflns	1548
Obsdreflns	*2226*
Parameters	128
R_{int}	0.0705
$R[I>2\sigma(I)]^a$	0.0464
W[all data]R^b	0.1470
GOF on F^2	1.049

[a] Conventional R on F_{hkl}: $\Sigma||F_o|-|F_c||/\sigma|F_o|$. [b] Weighted R on $|F_{hkl}|^2$: $\Sigma[w(F_o^2-F_c^2)^2]/\Sigma[w(F_o^2)^2]^{1/2}$.

Table S2. The fluorescence intensity(a.u.) of probe L and L-Cu2+ complex *versus* different pH value within 1440mins (565nm).

	pH value	Time			
		0min	360min	720min	1440min
Probe L	3	385.5018	383.2117	381.1152	380.0759
	4	382.6145	381.1132	380.0795	380.1572
	5	385.8454	385.0024	384.157	383.2157
	6	383.1325	382.154	381.6548	380.1422
	7	384.446	384.1243	381.1546	380.0154
	8	384.8546	385.0154	382.145	379.6841
	9	381.1106	381.0012	379.4952	375.8647
	10	381.6832	380.1452	379.4584	379.1452
	11	381.3092	380.145	378.2168	379.1445
L- Cu2+ complex	3	273.7491	272.144	270.1447	269.4473
	4	245.9818	245.0147	243.2589	241.3647
	5	231.7205	230.1747	229.3477	224.6647
	6	219.3097	218.1111	217.567	215.4468
	7	155.5946	155.3174	151.1577	149.5747
	8	45.8488	44.1113	42.4973	40.4178
	9	35.51372	33.479	31.409	30.1479
	10	35.58966	34.1475	33.247	30.0048
	11	21.48677	20.1398	17.0143	17.1148

Table S3. Comparison data with reported Cu^{2+} sensors

Structure	One-step synthesis	Fluorescence	solvent	Detection limit
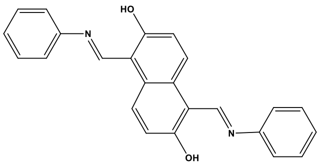 **Our work**	Yes	Turn-off	THF/ H20 (4:1)	0.0164μM
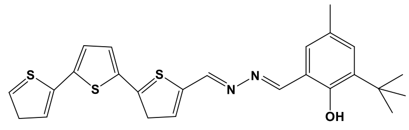 **3TDB[1]**	No	Turn-off	DMSO/H20 (1:1)	0.42μM
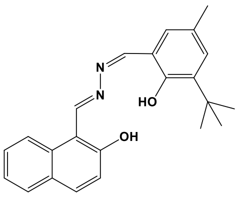 **ADC[2]**	Yes	Turn-off	THF/ H20 (1:1)	1.6μM
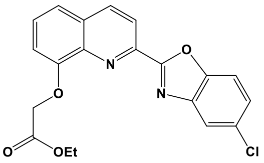 **Probe 1[3]**	No	Turn-off	EtOH/H20 (1:9)	0.27μM
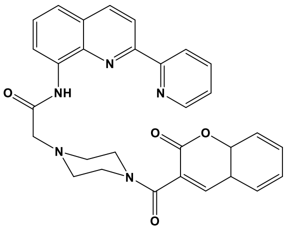 **CPC[4]**	No	Turn-off	MeCN/ H20 (2:1)	0.46μM

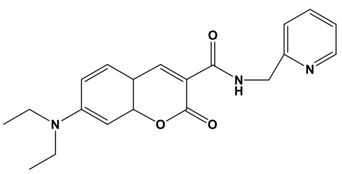 **Probe 1[5]**	No	Turn-off	DMSO/H_20 (1:9)	0.5μM
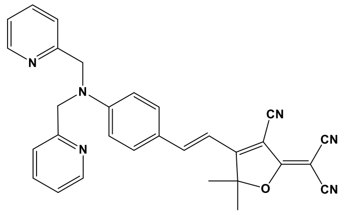 **BT[6]**	Yes	Turn-off	EtOH/ H_20 (1:4)	1μM
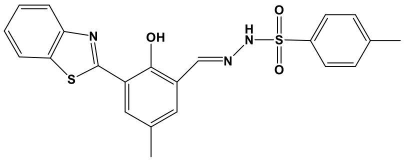 **BHMB[7]**	No	Turn-off	DMSO/H_20 (3:7)	0.047μM
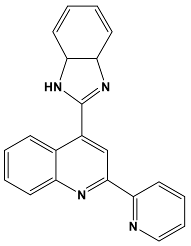 **ImPQ[8]**	No	Turn-off	MeCN/ H_20 (9:1)	1.86μM

Reference

1. Guo, Z.; Hu, T.; Wang, X.; Sun, T.; Li, T.; Niu, Q., Highly sensitive and selective fluorescent sensor for visual detection of Cu2+ in water and food samples based on oligothiophene derivative. *Journal of Photochemistry and Photobiology A: Chemistry* **2019,** *371*, 50-58.
2. Niu, Q.; Sun, T.; Li, T.; Guo, Z.; Pang, H., Highly sensitive and selective colorimetric/fluorescent probe with aggregation induced emission characteristics for multiple targets of copper, zinc and cyanide ions sensing and its practical application in water and food samples. *Sensors and Actuators B: Chemical* **2018,** *266*, 730-743.
3. Han, J.; Tang, X.; Wang, Y.; Liu, R.; Wang, L.; Ni, L., A quinoline-based fluorescence "on-off-on" probe for relay identification of Cu2+ and Cd2+ ions. *Spectrochimica Acta Part A: Molecular and Biomolecular Spectroscopy* **2018,** *205*, 597-602.
4. Zhang, Y.; Guo, X.; Tian, X.; Liu, A.; Jia, L., Carboxamidoquinoline–coumarin derivative: a ratiometric fluorescent sensor for Cu (II) in a dual fluorophore hybrid. *Sensors and Actuators B: Chemical* **2015,** *218*, 37-41.

5. Jung, H. S.; Kwon, P. S.; Lee, J. W.; Kim, J. I.; Hong, C. S.; Kim, J. W.; Yan, S.; Lee, J. Y.; Lee, J. H.; Joo, T., Coumarin-derived Cu2+-selective fluorescence sensor: synthesis, mechanisms, and applications in living cells. *Journal of the American Chemical Society* **2009,** *131* (5), 2008-2012.
6. Chen, D.; Chen, P.; Zong, L.; Sun, Y.; Liu, G.; Yu, X.; Qin, J., Colorimetric and fluorescent probes for real-time naked eye sensing of copper ion in solution and on paper substrate. *Royal Society open science* **2017,** *4* (11), 171161.
7. Zeng, S.; Li, S.-J.; Sun, X.-J.; Liu, T.-T.; Xing, Z.-Y., A dual-functional chemosensor for fluorescent on-off and ratiometric detection of Cu2+ and Hg2+ and its application in cell imaging. *Dyes and Pigments* **2019,** *170*, 107642.
8. More, P. A.; Shankarling, G. S., Reversible 'turn off'fluorescence response of Cu2+ ions towards 2-pyridyl quinoline based chemosensor with visible colour change. *Sensors and Actuators B: Chemical* **2017,** *241*, 552-559.
